# Anonymous and Efficient Chaotic Map-Based Authentication Protocol for Industrial Internet of Things

**DOI:** 10.3390/s25247676

**Published:** 2025-12-18

**Authors:** Dake Zeng, Akhtar Badshah, Shanshan Tu, Xin Ai, Hisham Alasmary, Muhammad Waqas, Muhammad Taimoor Khan

**Affiliations:** 1 College of Computer Science, Beijing University of Technology, Beijing 100124, China; pete.zeng@akuhome.com (D.Z.); sstu@bjut.edu.cn (S.T.); aixin1022@gmail.com (X.A.); 2 Department of Software Engineering, University of Malakand, Dir Lower, Chakdara 18800, Pakistan; 3 Department of Computer Science, College of Computer Science, King Khalid University, Abha 61421, Saudi Arabia; alasmary@kku.edu.sa; 4 School of Computing and Mathematical Sciences, Faculty of Engineering and Science, University of Greenwich, London SE10 9LS, UK; m.khan@greenwich.ac.uk

**Keywords:** Industrial Internet of Things, security, privacy, authentication, key agreement, biometrics

## Abstract

The exponential growth of Internet infrastructure and the widespread adoption of smart sensing devices have empowered industrial personnel to conduct remote, real-time data analysis within the Industrial Internet of Things (IIoT) framework. However, transmitting this real-time data over public channels raises significant security and privacy concerns. To prevent unauthorized access, user authentication mechanisms are crucial in the IIoT environment. To mitigate security vulnerabilities within IIoT environments, a novel user authentication and key agreement protocol is proposed. The protocol is designed to restrict service access exclusively to authorized users of designated smart sensing devices. By incorporating cryptographic hash functions, chaotic maps, Physical Unclonable Functions (PUFs), and fuzzy extractors, the protocol enhances security and functional integrity. PUFs provide robust protection against tampering and cloning, while fuzzy extractors facilitate secure biometric verification through the integration of smart cards, passwords, and personal biometrics. Moreover, the protocol accommodates dynamic device enrollment, password and biometric updates, and smart card revocation. A rigorous formal security analysis employing the Real-or-Random (ROR) model was conducted to validate session key security. Complementary informal security analysis was performed to assess resistance to a broad spectrum of attacks. Comparative performance evaluations unequivocally demonstrate the protocol’s superior efficiency and security in comparison to existing benchmarks.

## 1. Introduction

The Industrial Internet of Things (IIoT) employs numerous devices for real-time data sensing, transmission, and analysis, improving industrial control, productivity, and product quality while reducing costs and resource consumption. However, as IIoT expands with Industry 5.0, the rapid proliferation and interaction of IoT devices introduce critical security challenges [1,2,3,4]. Many IIoT devices incorporate lightweight software and hardware to minimize costs, limiting their ability to support robust security comparable to traditional Internet environments. This limitation creates vulnerabilities, making devices prone to attacks. Furthermore, deployment in remote, unmonitored environments exacerbates risks, exposing critical industrial processes to malicious disruptions [5,6,7].

In general, a typical IIoT system comprises smart sensing devices, such as temperature and humidity sensors, vibration sensors, and RFID tags, alongside users and gateway nodes, collectively forming a wireless sensor network (WSN) [8,9,10]. However, the dependence on wireless communication technologies exposes IIoT systems to substantial security vulnerabilities, as malicious adversaries can compromise system security through attacks such as eavesdropping and message tampering [11,12,13]. Authentication and key agreement mechanisms are regarded as one of the most effective countermeasures against these threats [14,15,16]. Nevertheless, given the intrinsic resource limitations of sensing devices, authentication protocols deployed in such settings must carefully balance computational efficiency with robust security guarantees.

### 1.1. Existing Research and Motivation

Recently, numerous research efforts have focused on developing anonymous and lightweight authenticated key agreement protocols, aiming to enhance security, privacy, and efficiency in IIoT environments [17,18,19,20]. Turkanović et al. [21] proposed a mutual authentication scheme that relied on a pre-shared cryptographic key between the sensor node and the user. Their scheme employed simple hash and XOR operations to accommodate the resource constraints of WSN. However, a subsequent analysis by Tai et al. [22] demonstrated that the protocol developed by Turkanović et al. fails to provide anonymity and is susceptible to sensor-capture attacks. Chen et al. [23] introduced an authentication and key agreement protocol for industrial control systems. Nevertheless, their proposed solution suffers from high computational and communication overheads, susceptibility to ephemeral secret leakage (ESL) attacks, and a lack of perfect forward secrecy. Shuai et al. [24] proposed an authentication protocol utilizing the Rabin cryptosystem. However, their protocol remains vulnerable to offline guessing, user impersonation, privileged insider, eavesdropping, and stolen smart card attacks. Gong et al. [25] propose a certificateless anonymous mutual authentication scheme ensuring privacy, traceability, and scalability for IoT. The schemes presented in [26,27] rely on a clock synchronization assumption, limiting their applicability in practical IIoT deployments. Zhai et al. [28] proposed a lightweight authentication protocol that combines blockchain with chaotic maps to enable mutual authentication between smart devices and edge gateways in IIoT systems. However, their protocol relies solely on the security of the chaotic-map discrete logarithm problem and fails to provide anonymity and untraceability. Aman et al. [29] proposed a PUF-based device authentication protocol for IoT systems. However, subsequent analyses demonstrated that the protocol is susceptible to replay attacks and non-invasive physical attacks [30], and it does not account for the influence of environmental noise on PUF responses.

Rafique et al. [31] addressed a critical challenge in the IIoT concerning secure data transmission. Their research proposed a multifactor authentication key agreement protocol that balanced robust security with resource limitations. This protocol utilized bitwise XOR, cryptographic hash functions, and symmetric cryptography to create a secure system tailored for resource-constrained environments, ensuring high-level security while enabling remote access to sensing devices. However, ref. [32] identified that Rafique’s protocol is vulnerable to attacks involving the loss of smart cards or devices. Eldefrawy et al. [33] proposed a user authentication method for IIoT systems, focusing on computational and communication efficiency. While this protocol was efficient, it did not provide mutual authentication between users and the smart devices or sensor nodes within the system. Harishma et al. [34] developed a method for securing data transmission in cyber-physical systems with heterogeneous components. Nevertheless, their approach was found vulnerable to the ESL attack under the Canetti and Krawczyk (CK) adversary model [35]. Additionally, the protocol did not support the dynamic incorporation of new IoT smart devices, limiting its practical application. Chen et al. [36] designed a key agreement and user authentication system for IoT environments. Although their protocol was efficient in terms of computational and communication costs, it was vulnerable to insider attacks, node-capturing attacks, and gateway node-bypassing attacks, and lacked untraceability [37,38,39].

In summary, although several commendable AKA protocols have been proposed for IIoT systems, most remain impractical for deployment on resource-constrained smart sensing devices due to their substantial resource overhead [40,41,42]. Furthermore, their incomplete security and functional guarantees further diminish their applicability in real-world deployments. Addressing these limitations is critical to enabling the efficient and secure operation of IIoT environments. The comparative summary is given in Table 1.

### 1.2. Contribution

This paper proposes an anonymous and efficient Chaotic Map-based authentication protocol for the IIoT environment that ensures both efficiency and security. The main contributions are as follows:

1. We propose a novel chaotic map-based mutual authentication and session key agreement protocol for the IIoT environment, where independent session keys are generated between users and smart sensing devices to ensure secure communications.
2. We design the proposed protocol by integrating a one-way cryptographic hash function, chaotic map, physical unclonable function (PUF), and fuzzy extractor to enhance security and functional integrity. The PUF component provides robust protection against tampering and cloning attacks on the smart sensing device.
3. We conduct a formal security analysis of the protocol using the real-or-random (ROR) model to rigorously assess and ensure session key security. Additionally, we provide an informal security analysis to demonstrate resistance to a broad spectrum of attacks.
4. A rigorous comparative performance evaluation was conducted to assess the security, functionality, communication overhead, and computational efficiency of the proposed protocol in relation to existing benchmarks. The study clearly demonstrates the proposed protocol’s superior efficiency and enhanced security features compared to existing protocols.

### 1.3. Novelty

The proposed protocol introduces several key innovations that collectively address critical gaps in existing IIoT authentication protocols. First, it pioneers a hybrid security architecture that uniquely integrates authentication based on chaotic-map and lightweight cryptographic hashing with PUF-based device identity verification, achieving both algorithmic robustness and hardware-rooted trust. Unlike existing protocols that rely solely on lightweight cryptography or PUF, the proposed protocol further incorporates a fuzzy extractor to mitigate the impact of environmental disturbances on PUF responses in industrial settings. In addition, the protocol establishes an independent session key directly between the user and the device without involving a central entity, ensuring genuine end-to-end confidentiality for sensitive information. By avoiding the use of timestamps for replay protection, it also eliminates the dependence on strict clock synchronization. Finally, under a unified framework, the proposed protocol achieves the most comprehensive set of security features with exceptionally high efficiency, as demonstrated in its multidimensional comparison with existing protocols in terms of computational, runtime, communication, and storage overhead, an advantage unmatched by prior works. These contributions collectively position the proposed protocol as a holistic authentication framework expressly tailored to the distinct security and performance demands of IIoT environments.

### 1.4. Paper Organization

The remainder of this paper is organized as follows: Section 2 presents the background, including network and threat models, and foundational concepts. The proposed protocol is detailed in Section 3. A comprehensive security analysis is provided in Section 4 and formal security analysis is presented in Section 5. Section 6 offers a performance comparison with existing state-of-the-art protocols. Finally, the paper is concluded in Section 7.

## 2. Background

This section provides a comprehensive overview of the authentication model, threat model, and cryptographic foundations.

### 2.1. System Models

This section delineates the authentication model and threat models employed in the design of the proposed authentication and key agreement protocol.

#### 2.1.1. Authentication Model

**Figure 1:**
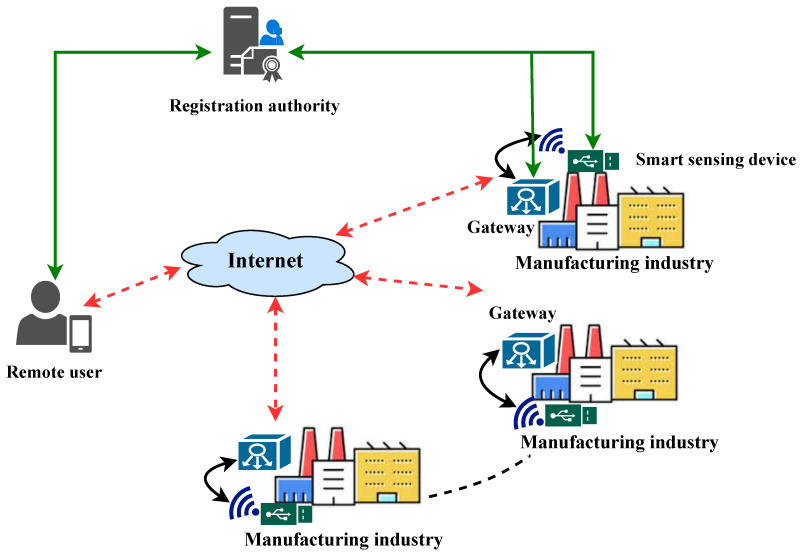
IoT-based industrial monitoring system architecture.

As illustrated in Figure 1, the proposed Internet of Things (IoT)-based smart sensing system for industrial monitoring aims to establish intelligent factories through the integration of IoT sensors and a robust authentication framework. This integrated system is designed to optimize supply chain, production, safety, and energy management. To address the challenges of securing real-time data transmission from resource-constrained IoT devices operating in inherently insecure wireless environments, a comprehensive authentication model is essential. This model safeguards data integrity and confidentiality while enabling authorized industrial personnel to securely access and utilize real-time data from smart sensing devices. A trusted registration authority (RA) establishes secure communication by registering all network entities and issuing cryptographic credentials. Authorized users, authenticated by the gateway node, can access the collected data through these gateway nodes, ensuring secure and reliable data transmission. The subsequent sections detail the proposed authentication protocol, which provides a secure and efficient mechanism for user authentication and data protection within the smart manufacturing environment [43,44,45].

#### 2.1.2. Threat Model

To assess the security of the proposed protocol, this study adopts the widely recognized Dolev-Yao (DY) threat model [46,47]. Within this framework, an adversary A is endowed with the capability to intercept, modify, delete, or inject spurious messages during communication over an insecure channel. Given the inherently hostile nature of the IIoT environment, IoT sensing devices are susceptible to physical compromise by A, both internal and external. Such breaches can lead to the unauthorized acquisition of sensitive credentials stored within these devices. Furthermore, an A may physically acquire an authorized user’s smart card, thereby enabling sophisticated power analysis attacks to compromise the stored secret credentials. Leveraging these credentials, the A can potentially extract sensitive user data, including identity, password, and biometric information. This compromised position facilitates a range of attack vectors, such as privileged insider attacks, replay attacks, man-in-the-middle (MitM) attacks, and impersonation attacks. Recently, the CK adversary model [48,49,50] has emerged as the *de facto* standard for evaluating the security of authenticated key agreement protocols. This model extends the DY adversary’s capabilities by granting the attacker the power to compromise secret credentials, session states, and session keys. Consequently, a robust user authentication protocol for the IIoT must safeguard against the exposure of sensitive information, limiting the adversary’s ability to compromise additional credentials even if some secrets are compromised. In addition, gateway nodes are protected by hardware security boundaries (for example, HSM/TEE) to safely store $\left\{ID_{GWN_{k}},X_{GWN_{k}}\right\}$. However, their databases are only semi-trusted and may be subject to physical capture attacks, allowing A to extract the hashed credentials stored within them. The symbols used in this paper and respective description is given in Table 2.

### 2.2. Preliminaries

This section provides a foundational overview of relevant cryptographic primitives, including PUFs, fuzzy extractors, and chaotic maps.

#### 2.2.1. Physical Unclonable Function

The PUF is a cryptographic mechanism that relies on challenge-response pairs (CRPs). The core process of a PUF involves generating a unique response (output) when given a specific challenge (input) [51,52], which can be formalized with $DR_{j}=PUF_{j}\left(C_{j}\right)$. Here, $C_{j}$ denotes the unique challenge presented to different devices ($SD_{j}$), while $DR_{j}$ signifies the corresponding unique response. PUFs function as irreversible mathematical functions, deriving cryptographic secrets from inherent physical variations found in integrated circuits (ICs). These variations, stemming from the manufacturing process, ensure that each IC generates a unique response to specific challenges. PUFs provide a cost-effective solution with minimal computational requirements and energy consumption, making them highly suitable for lightweight and physically secure cryptographic applications. They are advanced circuit primitives used to generate secret keys for cryptographic operations. For example, the Static Random Access Memory (SRAM) PUF is widely adopted due to SRAM’s critical role in electronic control units (ECUs). The SRAM PUF exploits random disparities in SRAM threshold voltages to establish a distinctive digital fingerprint for each device. Unlike traditional methods of key storage, the secret key is dynamically regenerated from the SRAM PUF within a secure environment. This approach ensures that even in the event of a memory breach, the secret key remains protected and immune to compromise. Importantly, any attempt to manipulate or tamper with a PUF alters the device’s behavior, thereby invalidating the PUF and facilitating tamper detection. However, in real-world noisy environments, factors such as temperature variations, voltage fluctuations, and device aging may cause a PUF to produce different responses even when supplied with the same challenge, which can result in the erroneous rejection of secret parameters generated solely from PUF responses during authentication. To address this important issue, we integrate a fuzzy extractor with the PUF. During the registration phase, the device first generates a response $DR_{j}=PUF_{j}\left(C_{j}\right)$. Subsequently, in an authentication session, the device regenerates a new response $DR_{j}^{\prime }=PUF_{j}\left(C_{j}\right)$. When the Hamming distance $DIST(DR_{j},DR_{j}^{\prime })$ falls within the tolerable error threshold *t*, the fuzzy extractor is used to reconcile $\left(DR_{j},DR_{j}^{\prime }\right)$, thereby ensuring stable key derivation.

#### 2.2.2. Fuzzy Extractors

Fuzzy extractors are cryptographic mechanisms designed to derive stable and secure cryptographic keys from inherently noisy data sources, such as biometric measurements or PUFs [53]. By mitigating the impact of noise and variations inherent in these data types, fuzzy extractors enhance the robustness and security of recognition systems. A fuzzy extractor consists of two primary components:

1. **Generation**$\left(Bk_{i},s_{i}\right)=Gen\left(B_{i}\right)$: Given a unique biometric or PUF measurement $B_{i}$ of entity *i*, the generation algorithm produces a cryptographic key $Bk_{i}$ and public auxiliary data $s_{i}$.
2. **Reproduction**$\;Bk_{i}^{\prime }=\mathrm{Rep}\left(B_{i}^{\prime },s_{i}\right)$: Utilizing the public auxiliary data $s_{i}$ and a noisy measurement $B_{i}^{\prime }$ approximating $Bk_{i}$, the reproduction algorithm reconstructs the original key $Bk_{i}^{\prime }$. For sufficiently similar input measurements (typically within a predetermined Hamming distance), $Bk_{i}^{\prime }$ will be identical to $Bk_{i}$.

#### 2.2.3. Chaotic Map

Chaotic map encryption leverages the intrinsic unpredictability and extreme sensitivity to initial conditions of chaotic systems for cryptographic applications [54,55,56]. This method employs the enhanced Chebyshev polynomial, defined as $T_{n}\left(x\right)=2xT_{n-1}\left(x\right)-T_{n-2}\left(x\right)\;\mathrm{mod}\;lp$, where lp is a large prime number, n≥2, and x∈(−∞,∞). Alternatively, $T_{n}\left(x\right)=\cos\left(n\cdot \arccos\left(x\right)\right)$. The semigroup property of Chebyshev polynomials, expressed as $T_{n}\left(T_{m}\left(x\right)\right)=T_{m}\left(T_{n}\left(x\right)\right)=T_{mn}\left(x\right)\;\mathrm{mod}\;lp$, forms the basis for the encryption process. The security of this encryption scheme relies on the computational intractability of the chaotic map discrete logarithm problem (CMDLP). An adversary’s ability to solve this problem within a given time frame is quantified as $Adv_{A}^{CMDLP}\left(rt\right)=Pr\left[A\left(x_{1},x_{2}\right)=m|T_{m}\left(x_{1}\right)=x_{2}\;\mathrm{mod}\;lp\right]$, where $Z_{p}^{*}=\left\{1,2,\ldots ,p-1\right\}$ and $r\in Z_{p}^{*}$. Some existing studies [57,58,59] indicate that, A may be able to solve the CMDLP problem in polynomial time, thereby compromising the security of cryptographic systems based on Chebyshev polynomials. However, this attack capability requires A to obtain both $T_{m}\left(x\right)$ and *x* simultaneously. To counteract this, the proposed protocol maintains the encryption of *x* during transmission. The protocol is grounded in the computational Diffie-Hellman (CDH) problem adapted to the chaotic map domain. Even with knowledge of $T_{m}\left(x\right)$ and $T_{n}\left(x\right)$, computing $T_{m}\left(T_{n}\left(x\right)\right)=T_{n}\left(T_{m}\left(x\right)\right)\;\mathrm{mod}\;lp$ remains computationally infeasible for an adversary, providing the cryptographic strength of the system.

### 2.3. Design Objectives

The protocol aims to achieve the following design goals:

- **Anonymity:** Protect user privacy by ensuring that their identities cannot be inferred from messages, maintaining anonymity throughout communications.
- **Mutual Authentication:** Verify the authenticity of users, gateways, and smart sensing devices to establish trust within the network.
- **Session Key Agreement:** Securely establish session keys between users and smart sensing devices, independent of the gateway, for protected communication.
- **Unlinkability:** Prevent the correlation of intercepted messages with specific users, preserving message anonymity.
- **Forward Security:** Protect the confidentiality of past communications by ensuring the compromise of current session keys does not affect the security of previous sessions.
- **Resistance to Common Attacks:** Strengthen the protocol’s defenses against replay, offline password guessing, and impersonation attacks to enhance overall IIoT network security.

## 3. The Proposed Protocol

This section presents a secure protocol tailored for IIoT environments to guarantee that only authorized users can access smart sensing devices. The protocol incorporates SHA-256 hashing, XOR operations, and PUFs to safeguard smart sensing devices against physical tampering. Furthermore, to address replay attacks, the protocol avoids reliance on clock synchronization among network entities. The subsequent subsections outline the various phases of the proposed protocol.

### 3.1. Pre-Deployment Phase

In this phase, the RA is responsible for registering the GWNs and smart sensing devices prior to their deployment. A secure channel is assumed for this one-time setup, as commonly adopted in IIoT protocols, typically ensured through administrator-supervised or trusted in-person registration.

#### 3.1.1. GWN Registration

The RA performs the following operations to register a GWN denoted as $GWN_{k}$.

1. Step GR-1: The RA selects a distinct and unique identity, $ID_{GWN_{k}}$, and a secret parameter, $X_{GWN_{k}}$. These are then transmitted securely to $GWN_{k}$ through a secure channel.
2. Step GR-2: $GWN_{k}$ securely stores the received parameters, $\left\{ID_{GWN_{k}},X_{GWN_{k}}\right\}$, in its secure database.

#### 3.1.2. Smart Sensing Device Registration

To register a smart sensing device, such as $SD_{j}$, the RA follows these steps:

1. Step DR-1: The RA starts the registration process by generating a unique challenge parameter, $C_{j}$. This parameter is securely transmitted to $SD_{j}$ over a secure channel.
2. Step DR-2: Upon receiving $C_{j}$ from the RA, $SD_{j}$ uses a PUF(·) to generate the response parameter $DR_{j}$. This response is then securely sent back to the RA.
3. Step DR-3: The RA selects an identity $ID_{j}$ and sends the pair $C_{j},DR_{j}$ to the appropriate gateway node $GWN_{k}$ through a secure channel.
4. Step DR-4: Upon receiving the $\left\{ID_{j},C_{j},DR_{j}\right\}$ from the RA, $GWN_{k}$ generates a random number $r_{j}$ and computes $X_{j}=h(ID_{j}∥X_{GWN_{k}}∥r_{j})$. It then stores the parameters $\left\{ID_{j},\;r_{j},\;C_{j},DR_{j}\right\}$ for $SD_{j}$ in its database and sends $X_{j}$ back to the RA through a secure channel.
5. Step DR-5: The RA finally transmits the parameters $\left\{ID_{j},\;X_{j}\right\}$ to $SD_{j}$, which then stores these parameters in its memory.

#### 3.1.3. User Registration

To ensure secure communication with an SD that has been accessed, $U_{i}$ must securely register at the RA through the following steps.

1. Step UR-1: User $U_{i}$ submits their registration request to the RA, along with their identity $ID_{i}$, through a secure channel.
2. Step UR-2: The RA then assesses whether the request originates from a legitimate user. It computes $h(ID_{i}∥X_{RA}∥T_{i})$ using a hash function, where $X_{RA}$ is the master secret key of the RA and $T_{i}$ is the registration timestamp. Based on the computed hash, it consults the database for the corresponding status. If the status indicates that the user is already registered (status=1), the registration process ceases. Otherwise, the RA proceeds with registration and picks a unique temporary identity $TID_{i}$, a unique pseudo-identity $PID_{i}$, and a random number $r_{i}$. The user’s smart card $SC_{i}$ is configured with <$TID_{i},r_{i},PID_{i},SCN_{i},h\left(\cdot \right)$>. The RA stores <$ID_{i},SCN_{i},T_{i},[h(ID_{i}∥X_{RA}∥T_{i}),\mathrm{status}]$> in its database and forwards the following parameters to the corresponding gateway node $GWN_{k}$ as <$TID_{i}^{o}=TID_{i},\;TID_{i}^{n}=TID_{i},\;PID_{i},\;r_{i},\;SCN_{i}$> via a secure channel and dispatches $SC_{i}$ to $U_{i}$.
3. Step UR-3: Upon receiving <$TID_{i}^{o}=TID_{i},TID_{i}^{n}=TID_{i},PID_{i},r_{i},SCN_{i}$> from the RA, $GWN_{k}$ computes $X_{i-g}=\left(PID_{i}∥r_{i}\right)⊕h\left(SCN_{i}∥X_{GWN_{k}}\right)$. $GWN_{k}$ then stores the parameters as <$TID_{i}^{o}=TID_{i},TID_{i}^{n}=TID_{i},X_{i-g},SCN_{i}$> in its database.
4. Step UR-4: Upon receipt, $U_{i}$ inserts $SC_{i}$ into a card reader, reads the biometric data $B_{i}$, and enters their $ID_{i}$ and password $PW_{i}$ and picks a random number $n_{i}$. The smart card then proceeds to compute the following:
  $$\begin{array}{cc} \left(Bk_{i},s_{i}\right) & =\mathrm{Gen}\left(B_{i}\right),X_{i}=\left(s_{i}∥n_{i}\right)⊕h\left(ID_{i}∥PW_{i}\right)\\ Auth_{i} & =h\left(ID_{i}∥PW_{i}∥Bk_{i}∥n_{i}\right)\\ Y_{i} & =\left(PID_{i}∥r_{i}\right)⊕h\left(ID_{i}∥PW_{i}∥Bk_{i}\right) \end{array}$$
  where Gen(·) is a fuzzy extractor generator function and $Auth_{i}$ is an authentication parameter. Finally, the smart card replaces the parameters <$r_{i},PID_{i}$> with <$X_{i},Y_{i},Auth_{i}$> and stores <$TID_{i},X_{i},Y_{i},Auth_{i},SCN_{i},h\left(\cdot \right),\mathrm{Gen}\left(\cdot \right),\mathrm{Rep}\left(\cdot \right)$>.

### 3.2. Login Phase

A legitimate user, for instance, $U_{i}$, must first authenticate themselves using their credentials and smart card. The specific steps are as follows:

1. Step L-1: User $U_{i}$ inserts their designated smart card and subsequently enters their unique identity ($ID_{i}^{\prime }$), password ($PW_{i}^{\prime }$), and provides biometric data ($B_{i}^{\prime }$) via the designated sensor.
2. Step L-2: Based on the provided inputs, the following computations are carried out:
  $$\begin{array}{cc} \left(s_{i}^{\prime }\|n_{i}^{\prime }\right) & =X_{i}⊕h\left(ID_{i}^{\prime }\|PW_{i}^{\prime }\right),Bk_{i}^{\prime }=\mathrm{Rep}\left(B_{i}^{\prime },s_{i}^{\prime }\right)\\ Auth_{i}^{\prime } & =h(ID_{i}^{\prime }\|PW_{i}^{\prime }\|Bk_{i}^{\prime }\|n_{i}^{\prime }) \end{array}$$
  The $Auth_{i}^{\prime }$ is verified against $Auth_{i}$ as follows: $Auth_{i}^{\prime }\overset{?}{=}Auth_{i}$ If the verification fails, the login procedure is aborted. If the verification succeeds, the sign-in process is considered successful, and the following parameters are computed: $\left(PID_{i}∥r_{i}\right)=Y_{i}⊕h(ID_{i}^{\prime }∥PW_{i}^{\prime }∥Bk_{i}^{\prime })$.

### 3.3. Authentication and Key Agreement Phase

**Figure 2:**
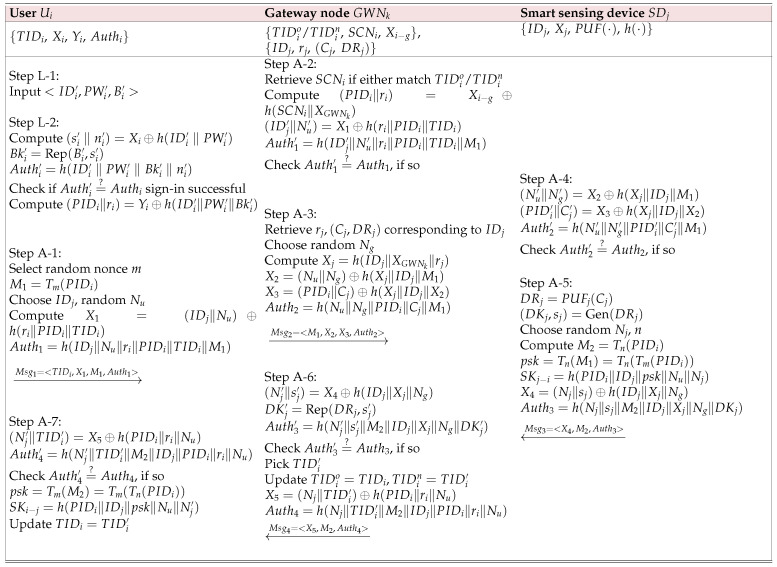
Login and authentication phases.

The following steps are essential for completing this phase, as illustrated in Figure 2.

1. Step A-1: $U_{i}$ generates a random nonce *m* and uses it to compute $M_{1}=T_{m}\left(PID_{i}\right)$ as a chaotic-map variable. Then, $U_{i}$ chooses $ID_{j}$ and selects random nonce $N_{u}$, and calculates $X_{1}=\left(ID_{j}∥N_{u}\right)⊕h(r_{i}∥PID_{i}∥TID_{i})$. Additionally, $U_{i}$ computes $Auth_{1}=h(ID_{j}\|N_{u}\|r_{i}\|PID_{i}\|TID_{i}\left\|M_{1}\right)$. Afterward, $U_{i}$ constructs $Msg_{1}=<TID_{i},\;X_{1},\;M_{1},\;Auth_{1}>$ and sends it to the gateway node $GWN_{k}$ through an open insecure channel.
2. Step A-2: Upon receiving $Msg_{1}$ from $U_{i}$, $GWN_{k}$ searches for $SCN_{i}$, which should match either $TID_{i}^{o}$ or $TID_{i}^{n}$. The following computations are then performed:
  $$\begin{array}{cc} \left(PID_{i}∥r_{i}\right) & =X_{i-g}⊕h\left(SCN_{i}∥X_{GWN_{k}}\right)\\ \left(ID_{j}^{\prime }∥N_{u}^{\prime }\right) & =X_{1}⊕h(r_{i}∥PID_{i}∥TID_{i})\\ Auth_{1}^{\prime } & =h(ID_{j}^{\prime }∥N_{u}^{\prime }∥r_{i}∥PID_{i}∥TID_{i}∥M_{1}) \end{array}$$
  The calculated $Auth_{1}^{\prime }$ is checked for equality with $Auth_{1}$, i.e., $Auth_{1}^{\prime }\overset{?}{=}Auth_{1}$. If they do not match, the process is terminated. If they match, the procedure continues to the next steps.
3. Step A-3: For $ID_{j}$, $GWN_{k}$ retrieves $r_{j},\left(C_{j},DR_{j}\right)$ and selects a random nonce $N_{g}$. The following computations are then performed:
  $$\begin{array}{cc} X_{j} & =h(ID_{j}∥X_{GWN_{k}}∥r_{j})\\ X_{2} & =\left(N_{u}∥N_{g}\right)⊕h(X_{j}∥ID_{j}∥M_{1})\\ X_{3} & =\left(PID_{i}∥C_{j}\right)⊕h(X_{j}∥ID_{j}∥X_{2})\\ Auth_{2} & =h(N_{u}∥N_{g}∥PID_{i}∥C_{j}∥M_{1}) \end{array}$$
  Finally, $GWN_{k}$ constructs $Msg_{2}$ as $Msg_{2}=<M_{1},X_{2},$$X_{3},\;Auth_{2}>$ and transmits it to $SD_{j}$ through an open insecure channel.
4. Step A-4: Upon receiving $Msg_{2}$ from $GWN_{k}$, $SD_{j}$ retrieves $ID_{j}$ and $X_{j}$ and performs the following:
  $$\begin{array}{cc} \left(N_{u}^{\prime }∥N_{g}^{\prime }\right) & =X_{2}⊕h(X_{j}∥ID_{j}∥M_{1})\\ \left(PID_{i}^{\prime }∥C_{j}^{\prime }\right) & =X_{3}⊕h(X_{j}∥ID_{j}∥X_{2})\\ Auth_{2}^{\prime } & =h(N_{u}^{\prime }∥N_{g}^{\prime }∥PID_{i}^{\prime }∥C_{j}^{\prime }∥M_{1}) \end{array}$$
  The resulting $Auth_{2}^{\prime }$ is compared with $Auth_{2}$ to verify if $Auth_{2}^{\prime }\overset{?}{=}Auth_{2}$. If they do not match, the process is aborted. If they match, the procedure proceeds to the next step.
5. Step A-5: $SD_{j}$ inputs the challenge $C_{j}$ into the PUF(·) function to obtain a device-specific but potentially noisy response, $DR_{j}=PUF_{j}\left(C_{j}\right)$. Due to the inherent variability of PUFs caused by environmental and hardware factors, $SD_{j}$ employs a fuzzy extractor to ensure reliable key derivation. Specifically, the fuzzy extractor generates a stable and reproducible secret key $DK_{j}$ and corresponding helper data $s_{j}$, denoted as $\left(DK_{j},s_{j}\right)=\mathrm{Gen}\left(DR_{j}\right)$. This process ensures that even if $DR_{j}$ slightly fluctuates under different conditions, the same $DK_{j}$ can be reliably recovered using the helper data during key reconstruction. Next, $SD_{j}$ selects a random integral string *n* and a random nonce $N_{j}$, and computes the following: $M_{2}=T_{n}\left(PID_{i}\right)$, $psk=T_{n}\left(M_{1}\right)=T_{n}\left(T_{m}\left(PID_{i}\right)\right)$, $SK_{j-i}=h(PID_{i}\|ID_{j}\left\|psk\right\|N_{u}\left\|N_{j}\right)$, $X_{4}=\left(N_{j}\|s_{j}\right)⊕h(ID_{j}\|X_{j}\left\|N_{g}\right)$, and $Auth_{3}=h(N_{j}\|s_{j}\|M_{2}\|ID_{j}\|X_{j}\|N_{g}\left\|DK_{j}\right)$, where $SK_{j-i}$ is the generated secret session key between $SD_{j}$ and $U_{i}$. Finally, $SD_{j}$ constructs $Msg_{3}$ as $Msg_{3}=\langle X_{4},\;M_{2},\;Auth_{3}\rangle$ and sends it to $GWN_{k}$ via an open insecure channel.
6. Step A-6: Upon receiving $Msg_{3}$ from $SD_{j}$, $GWN_{k}$ performs the following computations:
  $$\begin{array}{cc} \left(N_{j}^{\prime }∥s_{j}^{\prime }\right) & =X_{4}⊕h(ID_{j}∥X_{j}∥N_{g}),DK_{j}^{\prime }=\mathrm{Rep}\left(DR_{j},s_{j}^{\prime }\right)\\ Auth_{3}^{\prime } & =h(N_{j}^{\prime }∥s_{j}^{\prime }∥M_{2}∥ID_{j}∥X_{j}∥N_{g}∥DK_{j}^{\prime }) \end{array}$$
  $GWN_{k}$ accepts the message if $Auth_{3}^{\prime }\overset{?}{=}Auth_{3}$ holds true. Next, $GWN_{k}$ picks a unique temporary identity $TID_{i}^{\prime }$ and updates the old and new temporary identities as $TID_{i}^{o}=TID_{i}$ and $TID_{i}^{n}=TID_{i}^{\prime }$. Then, $GWN_{k}$ computes $X_{5}=\left(N_{j}∥TID_{i}^{\prime }\right)⊕h(PID_{i}∥r_{i}∥N_{u})$ and $Auth_{4}=h(N_{j}∥TID_{i}^{\prime }∥M_{2}∥ID_{j}∥PID_{i}∥r_{i}∥N_{u})$. Finally, $GWN_{k}$ sends $Msg_{4}=<X_{5},\;M_{2},\;Auth_{4}>$ to $U_{i}$ to $U_{i}$ via an open insecure channel.
7. Step A-7: Upon receiving $Msg_{4}$ from $GWN_{k}$, $U_{i}$ computes $\left(N_{j}^{\prime }∥TID_{i}^{\prime }\right)=X_{5}⊕h(PID_{i}∥r_{i}∥N_{u})$ and $Auth_{4}^{\prime }=h(N_{j}^{\prime }∥TID_{i}^{\prime }∥M_{2}∥ID_{j}∥PID_{i}∥r_{i}∥N_{u})$. $U_{i}$ accepts if $Auth_{4}^{\prime }\overset{?}{=}Auth_{4}$ holds true. Next, $U_{i}$ computes $psk=T_{m}\left(M_{2}\right)=T_{m}\left(T_{n}\left(PID_{i}\right)\right)$, $SK_{UC}=h(PID_{i}∥r_{i}∥N_{c}∥N_{u})$ and $SK_{i-j}=h(PID_{i}\|ID_{j}\left\|psk\right\|N_{u}\left\|N_{j}^{\prime }\right)$. Finally, $U_{i}$ stores the session key and updates the temporary identity as $TID_{i}=TID_{i}^{\prime }$.

### 3.4. Password and Biometrics Update Phase

The proposed protocol allows users to locally update their passwords without needing to interact with the RA. Users can modify both their password (${PW}_{i}$) and biometric information ($B_{i}$) by following these steps:

1. Step PBU-1: The legitimate user $U_{i}$ inserts their smart card and authenticates themselves with their credentials to update their password and biometrics. The following calculations are then performed using the provided inputs:
  $$\begin{array}{cc} \left(s_{i}^{\prime }\|n_{i}^{\prime }\right) & =X_{i}⊕h\left(ID_{i}^{\prime }\|PW_{i}^{\prime }\right),Bk_{i}^{\prime }=\mathrm{Rep}\left(B_{i}^{\prime },s_{i}^{\prime }\right)\\ Auth_{i}^{\prime } & =h(ID_{i}^{\prime }\|PW_{i}^{\prime }\|Bk_{i}^{\prime }\|n_{i}^{\prime }) \end{array}$$
  The $Auth_{i}^{\prime }$ is verified against $Auth_{i}$ as follows: $Auth_{i}^{\prime }\overset{?}{=}Auth_{i}$ If the verification fails, the login procedure is aborted. If the verification succeeds, $U_{i}$ can reset the password and update the biometrics data, and the following parameters are computed: $\left(PID_{i}∥r_{i}\right)=Y_{i}⊕h(ID_{i}^{\prime }∥PW_{i}^{\prime }∥Bk_{i}^{\prime })$.
2. Step PBU-1: Next, $U_{i}$ inputs new information ($PW_{i}^{n}$ and $B_{i}^{n}$), and the card executes the following computations:
  $$\begin{array}{cc} \left(Bk_{i}^{n},s_{i}^{n}\right) & =\mathrm{Gen}\left(B_{i}^{n}\right),X_{i}^{n}=\left(s_{i}^{n}∥n_{i}\right)⊕h\left(ID_{i}∥PW_{i}^{n}\right)\\ Auth_{i}^{n} & =h\left(ID_{i}∥PW_{i}^{n}∥Bk_{i}^{n}∥n_{i}^{n}\right)\\ Y_{i}^{n} & =\left(PID_{i}∥r_{i}\right)⊕h\left(ID_{i}∥PW_{i}^{n}∥Bk_{i}^{n}\right) \end{array}$$
  Subsequently, the system updates the existing parameters in the smart card $<\;TID_{i},\;X_{i},\;Y_{i},\;Auth_{i},$ $SCN_{i},\;h\left(\cdot \right),\;\mathrm{Gen}\left(\cdot \right),\;\mathrm{Rep}\left(\cdot \right)\;>$ with the new configurations $<TID_{i},\;X_{i}^{n},\;Y_{i}^{n},\;Auth_{i}^{n},\;SCN_{i},\;h\left(\cdot \right),\;\mathrm{Gen}\left(\cdot \right),$Rep(·)>.

### 3.5. Smart Card Revocation

The protocol enables smart card replacement without altering the user’s identity. If user $U_{i}$ loses or has their smart card stolen, they can request a replacement from the RA using the following procedure:

1. Step SCR-1: Initially, $U_{i}$ securely transmits their identity (${ID}_{i}$), credentials, and a replacement request to RA. The RA assesses these credentials and the validity of the request. Following confirmation, RA issues a replacement smart card, ${SCN}_{i}^{n}$, a unique pseudo-identity $PID_{i}^{n}$, and a random number $r_{i}^{n}$. Consequently, the freshly configured smart card $SC_{i}^{n}$ is configured with <$TID_{i}^{n},\;r_{i}^{n},\;PID_{i}^{n},\;SCN_{i}^{n},\;h\left(\cdot \right)$>. The RA stores <$ID_{i},\;SCN_{i}^{n},\;T_{i}^{n},\;[h(ID_{i}∥X_{RA}^{n}∥T_{i}^{n}),\;\mathrm{status}]$> in its database and forwards the following parameters to the corresponding gateway node $GWN_{k}$ as <$TID_{i}^{o}=TID_{i}^{n},$ $TID_{i}^{n}=TID_{i}^{n},\;PID_{i}^{n},\;r_{i}^{n},\;SCN_{i}^{n}$> via a secure channel and dispatches $SC_{i}^{n}$ to $U_{i}$.
2. Step SCR-2: Furthermore, upon receipt of the card, $U_{i}$ inserts it into a card reader and performs the same steps as outlined in Section 3.1.3 *(“User Registration”)*. Finally, the smart card stores $<TID_{i}^{n},\;X_{i}^{n},\;Y_{i}^{n},$ $Auth_{i}^{n},\;SCN_{i}^{n},\;h\left(\cdot \right),\;\mathrm{Gen}\left(\cdot \right),\;\mathrm{Rep}\left(\cdot \right)>$.

### 3.6. Dynamic Smart Device Addition Phase

This phase is essential for subsequent deployment of new smart sensing devices. To integrate a new device, denoted as $SD_{j}^{new}$, the RA performs the following offline steps:

1. Step DA-1: The RA starts the registration process by generating a unique challenge parameter, $C_{j}^{new}$. This parameter is securely transmitted to $SD_{j}^{new}$ over a secure channel.
2. Step DA-2: Upon receiving $C_{j}^{new}$ from the RA, $SD_{j}^{new}$ uses a PUF(·) to generate the response parameter $DR_{j}^{new}$. This response is then securely sent back to the RA.
3. Step DA-3: The RA selects an identity $ID_{j}^{new}$ and sends the pair $\left\{C_{j}^{new},DR_{j}^{new}\right\}$ to the appropriate gateway node $GWN_{k}$ through a secure channel.
4. Step DA-4: Upon receiving the $\left\{ID_{j}^{new},C_{j}^{new},DR_{j}^{new}\right\}$ from the RA, $GWN_{k}$ generates a random number $r_{j}^{new}$ and computes $X_{j}^{new}=h(ID_{j}^{new}∥X_{GWN_{k}}∥r_{j}^{new})$. It then stores the parameters $\left\{ID_{j}^{new},r_{j}^{new},C_{j}^{new},DR_{j}^{new}\right\}$ for $SD_{j}^{new}$ in its database and sends $X_{j}^{new}$ back to the RA through a secure channel.
5. Step DA-5: The RA finally transmits the parameters $\left\{ID_{j}^{new},X_{j}^{new}\right\}$ to $SD_{j}^{new}$, which then stores these parameters in its memory.

Once $SD_{j}^{new}$ is deployed, the $GWN_{k}$/RA will notify all registered users, enabling them to access services from $SD_{j}^{new}$ if needed.

## 4. Informal Security Analysis

In this section, security and functional attributes of the proposed protocol are examined through a descriptive analysis.

### 4.1. Smart Sensing Device Capture Attack

The physical unclonability of PUF technology safeguards smart sensing devices, such as $SD_{j}$, against unauthorized access. Any tampering with the PUF-based sensor will significantly alter its response or disable the device entirely. Consequently, extracting sensitive information from the PUF-equipped $SD_{j}$ becomes extremely challenging for potential attackers.

### 4.2. Gateway Node Capture Attack

Assume that A is a privileged insider within the IIoT system who is capable of physically compromising a gateway node and thereby obtaining the secret credentials stored on it, namely $\left\{TID_{i}^{o}/TID_{i}^{n},\;SCN_{i},\;X_{i-g}\right\}$ and $\left\{ID_{j},\;r_{j},\;\left(C_{j},\;DR_{j}\right)\right\}$, where $X_{i-g}=\left(PID_{i}∥r_{i}\right)⊕h\left(SCN_{i}∥X_{GWN_{k}}\right)$. However, due to the one-way property of the cryptographic hash function, the secret credentials $PID_{i}$ and $r_{i}$, which are bound to $U_{i}$, remain protected since $X_{GWN_{k}}$ is stored within a hardware-isolated region that is inaccessible to A. Moreover, $GWN_{k}$ does not store the identity $ID_{i}$, password $PW_{i}$, or biometric template $B_{i}$ of $U_{i}$. Given only $\left\{TID_{i}^{o}/TID_{i}^{n},\;SCN_{i}\right\}$ and the hashed credential $X_{i-g}$, even if A simultaneously acquires the user’s smart card, no valid information can be inferred that would compromise the security of the session key.

### 4.3. Anonymity and Untraceability

Considering the threat model described in Section 2.1.2, suppose that A intercepts the transmitted messages $Msg_{1}$ to $Msg_{4}$ during the login and authentication processes, as illustrated in Figure 2, over an insecure public channel. These intercepted messages contain secret random nonces, which are crucial for maintaining confidentiality. This confidentiality makes it extremely difficult for A to determine critical identifiers such as the user’s identity ($ID_{i}$), the user’s pseudo-identity ($PID_{i}$), or the smart sensing device’s identifier ($ID_{j}$). This mechanism ensures the anonymity of users and their associated smart sensing devices within the network. Additionally, the use of unique random nonces for each session prevents A from tracking users across different sessions. Even if A identifies the temporary identities of $U_{i}$ from the intercepted messages, the protocol’s design requires frequent renewal of these identifiers to new temporary ones ($TID_{i}^{n}$) with each session. This ensures that tracking users and devices over time is not possible. Consequently, the proposed protocol effectively guarantees the anonymity and untraceability of participants, establishing a robust security foundation within the system.

### 4.4. De-Synchronization Attack

In our protocol, users are assigned unique temporary identities ($TID_{i}$) during the registration phase. The gateway node ($GWN_{k}$) stores the parameters $<TID_{i}^{o}=TID_{i},TID_{i}^{n}=TID_{i},X_{i-g},SCN_{i}>$ for each user $U_{i}$. To counter de-synchronization attacks, the protocol maintains both the old and new temporary identities in the gateway node’s database, ensuring synchronization between $U_{i}$ and $GWN_{k}$. This design ensures the protocol’s functionality remains unaffected even if the final confirmation message is delayed or lost, thereby providing robust protection against de-synchronization attacks.

### 4.5. Replay Attack

The proposed protocol safeguards against replay attacks where an adversary A intercepts and attempts to reuse previously exchanged messages ($Msg_{1}$ to $Msg_{4}$), where $Msg_{1}=<TID_{i},\;X_{1},\;M_{1},\;Auth_{1}>$, $Msg_{2}=<M_{1},X_{2},$ $X_{3},\;Auth_{2}>$, $Msg_{3}=<X_{4},\;M_{2},\;Auth_{3}>$, and $Msg_{4}=<X_{5},\;M_{2},\;Auth_{4}>$. The strength of the protocol lies in its use of unpredictable, one-time random values (nonces) within each transmitted message. These nonces guarantee that each message is unique to a specific session, rendering them useless in any subsequent attempt by A to replay them. This effectively prevents replay attacks and ensures the protocol’s robustness.

### 4.6. MitM Attack

Even if an attacker (A ) intercepts messages ($Msg_{1}$ to $Msg_{4}$) intending to tamper with them and impersonate a user, the protocol’s design thwarts such attempts. Modifying the initial message ($Msg_{1}=<TID_{i},\;X_{1},\;M_{1},\;Auth_{1}>$) requires access to secret information like $N_{u}$, $ID_{j}$, and $PID_{i}$, which are beyond A’s reach. Similarly, for messages $Msg_{2}$, $Msg_{3}$, and $Msg_{4}$, the protocol relies on confidential data like $PID_{i}$, $DR_{j}$, $ID_{j}$, and others to generate valid nonces. Without this information, A cannot forge messages that appear legitimate. This ensures the protocol’s resistance to MitM attacks.

### 4.7. Mutual Authentication

The proposed protocol establishes mutual authentication between user $U_{i}$, gateway $GWN_{k}$, and sensor $SD_{j}$ via the subsequent three steps: (1) $GWN_{k}$ validates $U_{i}$ by checking $SCN_{i}$ and $Auth_{1}$; (2) $SD_{j}$ authenticates $GWN_{k}$ directly by verifying $Auth_{2}$ and indirectly by matching $PID_{i}$ from the transcripts with $U_{i}$; (3) $U_{i}$ verifies $Auth_{4}$ to confirm $GWN_{k}$ directly and also indirectly confirms $SD_{j}$, establishing the session key independently of the gateway node.

### 4.8. Session Key Security

The proposed protocol ensures session key security through a multi-layered approach. An adversary A cannot bypass local user authentication or access sensitive credentials such as $PID_{i}$ and $r_{i}$ stored on the smart card in encrypted form. This protection is due to the requirement of all three factors (password, biometric data, and smart card) for login. Additionally, the session key $SK_{j-i}=h(PID_{i}∥ID_{j}∥psk∥N_{u}∥N_{j})$ is derived using a combination of a one-way hash function and unique, secret parameters specific to each user, device, and communication session. These parameters include $PID_{i}$, psk, $N_{j}$, $N_{u}$, and $ID_{j}$. The one-way nature of the hash function ensures that even without knowledge of these secret parameters, deriving the session key remains computationally infeasible. This approach safeguards the confidentiality and integrity of communication within the protocol.

### 4.9. Perfect Forward Secrecy

In the proposed protocol, the session key $SK_{j-i}=h(PID_{i}∥ID_{j}∥psk∥N_{u}∥N_{j})$ established between $U_{i}$ and $SD_{j}$ is derived through a hybrid mechanism that combines random values, long-term secrets, and chaotic-map–based secrets, thereby ensuring perfect forward secrecy. First, these parameters are unique to each session and each communicating entity, preventing A from correlating information across different sessions or participants. Second, in every authentication session, the random values used to compute the chaotic-map encryption outputs are randomly selected, and $PID_{i}$ remains hidden from A. Due to the inherent hardness of the CMDLP, A can not derive $psk=T_{n}\left(T_{m}\left(PID_{i}\right)\right)$ that satisfies $M_{1}=T_{m}\left(PID_{i}\right)$ and $M_{2}=T_{n}\left(PID_{i}\right)$. Moreover, the random values and long-term secrets that contribute to $SK_{j-i}$ are protected by the cryptographic hash function and thus remain concealed from A. Overall, through this hybrid derivation mechanism that incorporates both long-term and ephemeral secrets, the proposed protocol ensures that the security of $SK_{j-i}$ does not rely on any single cryptographic component, thereby providing perfect forward secrecy.

### 4.10. ESL Attack

In the proposed protocol, the session key $SK_{j-i}=h(PID_{i}∥ID_{j}∥psk∥N_{u}∥N_{j})$ is generated between $U_{i}$ and $SD_{j}$ with the help of $GWN_{k}$. This key relies on session-specific and entity-specific secret information as well as random parameters. To assess the resilience of the key against ESL attacks, we examine two distinct scenarios:

1. *Scenario 1*: If the attacker A manages to obtain the temporary secrets $TID_{i}$, $N_{g}$, $N_{u}$, $N_{j}$, and psk, the hash function h(·)’s collision-resistant properties make it difficult for A to derive the session key without also having access to the long-term secrets $ID_{i}$, $PW_{i}$, $PID_{i}$, $ID_{j}$, $X_{j}$, and $DK_{j}$.
2. *Scenario 2*: Even if A gains possession of the long-term secrets $ID_{i}$, $PW_{i}$, $PID_{i}$, $ID_{j}$, $X_{j}$, and $DK_{j}$, the absence of short-term secrets still makes it impractical for A to compute the session key.

### 4.11. Stolen Smartcards and Privileged-Insiders Attacks

These scenarios explore the implications of a lost or stolen smartcard, $SC_{i}$, belonging to a registered user. The registration process commences with user $U_{i}$ transmitting $ID_{j}$ to the RA via a secure channel. Suppose a privileged-insider within the RA, denoted as adversary A, has access to the information $<TID_{i},r_{i},PID_{i},SCN_{i}>$. Subsequent to registration completion, it is assumed that adversary A has gained possession of user $U_{i}$’s stolen smartcard, denoted as $SC_{i}$ and then extracted the data $<TID_{i},X_{i},Y_{i},Auth_{i},SCN_{i}>$ from $SC_{i}$ using power analysis attacks. Extracting or guessing $ID_{i}$ from the values $\left(X_{i},Y_{i},Auth_{i}\right)$ without knowing $PW_{i}$ and $B_{i}$ of $U_{i}$ is computationally infeasible, as $PW_{i}$ and $B_{i}$ are protected by a collision-resistant hash function h(·). This demonstrates that identity guessing attacks are challenging for A. Similarly, deriving $PW_{i}$ from $\left(X_{i},Y_{i},Auth_{i}\right)$ without $ID_{i}$ and $B_{i}$ is computationally infeasible due to h(·)’s collision resistance. Additionally, it is computationally infeasible for the adversary A to derive $PW_{i}$ from the extracted parameters. Thus, the system is robust against offline password guessing attacks, even in the case of stolen smartcards or privileged insider threats.

### 4.12. Resistance to Modeling Attacks

The proposed protocol prevents modeling attacks by avoiding challenge-response transmission over insecure channels. Using collision-resistant hash function h(·) and XOR to encrypt secret parameters, adversary A cannot extract useful information from intercepted messages, ensuring security with minimal overhead.

### 4.13. Impersonation Attacks

To mitigate impersonation attacks, three scenarios are examined: *Case I* (*User Impersonation Attack*): Suppose an adversary A intercepts the message $Msg_{1}=<TID_{i},\;X_{1},\;M_{1},\;Auth_{1}>$. Using this information, A tries to impersonate the user by crafting a modified message $Msg_{1}^{A}=<TID_{i}^{A},\;X_{1}^{A},\;M_{1}^{A},\;Auth_{1}^{A}>$ to deceive others. However, without knowledge of $PID_{i}$, $r_{i}$, and $ID_{j}$, A cannot produce a valid $Msg_{1}$, rendering it incapable of establishing a credible communication with $GWN_{k}$ and thus preventing successful user impersonation.

*Case II* (*Gateway Node Impersonation Attack*): Consider the scenario where A intercepts $Msg_{2}=<M_{1},X_{2},$ $X_{3},\;Auth_{2}>$. To impersonate a gateway node, A would need to fabricate $Msg_{2}$ to convince a smart sensing device of its authenticity. However, without knowledge of $ID_{j}$ and $X_{j}$, A cannot compute $X_{2}$, $X_{3}$, and $Auth_{2}$. Therefore, A cannot create a valid $Msg_{2}$ to impersonate a gateway node, and similarly, A cannot compute $Msg_{4}$ to deceive $U_{i}$. Thus, A fails to impersonate a gateway node.

*Case III* (*Smart Sensing Device Impersonation Attack*): When A intercepts the message $Msg_{3}=<X_{4},M_{2},Auth_{3}>$ from $SD_{j}$ to $GWN_{k}$, impersonating $SD_{j}$ requires crafting a credible $Msg_{3}$. Nonetheless, lacking crucial information like $ID_{j}$, $X_{j}$, $DK_{j}$, $N_{j}$, $s_{j}$, $N_{g}$, and $PID_{i}$, A fails to generate a legitimate $Msg_{3}$. This inability to forge a valid message demonstrates the protocol’s effectiveness in thwarting attempts at impersonating the smart sensing device, underscoring its security against such types of attacks.

## 5. Formal Security Analysis

This section analyzes the security of the proposed protocol using the ROR model, formally proving session key security. Before presenting the session key proof (Theorem 1), we introduce essential ROR model primitives.

*Participants*. The proposed protocol involves three primary participants: the user $U_{i}$, the gateway node $GWN_{k}$, and the smart sensing device $SD_{j}$. Instances of these participants are represented as $\Omega_{U}^{t_{1}}$, $\Omega_{GWN}^{t_{2}}$, and $\Omega_{SD}^{t_{3}}$, corresponding to the $t_{1}$th instance of $U_{i}$, the $t_{2}$th instance of $GWN_{k}$, and the $t_{3}$th instance of $SD_{j}$, respectively. Each instance is treated as an oracle, and $\Omega^{t}$ is occasionally used to denote the *t*th instance of any given entity.

*Partnership*. Entities $\Omega^{t_{1}}$ and $\Omega^{t_{2}}$ in the protocol are considered partners if they meet the following criteria: (1) Both entities have reached an acceptance state; (2) They share the same session identifier SID; and (3) A session key has been established between them.

*Freshness*. An entity $\Omega^{t}$ is considered to be fresh if its established session key has not been compromised by the adversary A. An entity $\Omega^{t}$ is deemed fresh if its established session key remains uncompromised by the adversary A.

*Adversary*. In the ROR model, the adversary A’s capabilities are represented by conducting specific oracle queries: In the ROR model, the capabilities of the adversary A are represented through specific oracle queries defined in Table 3. It is important to emphasize that, while A is granted the ability to extract the secret credentials stored in a user’s smart card via the **$CorruptSmartcard(\Omega^{t})$** query, deriving either the long-term or ephemeral secrets from the hashed credentials remains well beyond A’s capability.

**Definition** **1** (Semantic Security). *In the context of the RoR model, the adversary A is tasked with distinguishing between the actual session key and a random string during the Test query. The adversary A attempts to correctly guess the value of b to succeed in this challenge. The advantage of the adversary in compromising the semantic security of the proposed protocol P is denoted as $Adv_{A}^{P}$ and is defined by the equation $Adv_{A}^{P}=2\cdot \mathrm{Pr}\left[b^{\prime }=b\right]-1$. If there exists a sufficiently small function ϵ such that $Adv_{A}^{P}<\epsilon$, then the proposed protocol P is considered to be semantically secure.*

**Definition** **2** (Security of PUF). *For two secure functions $PUF{\left(\cdot \right)}_{1}$ and $PUF{\left(\cdot \right)}_{2}$, given any inputs $c_{1}$ and $c_{2}$ in ${0,1}^{k}$, the probability that the Hamming distance $HD(PUF{\left(c_{1}\right)}_{1},PUF{\left(c_{2}\right)}_{2})$ exceeds d is 1−ϵ. Here, d represents the fault tolerance level.*

**Definition** **3** (CMDLP). *The advantage of an adversary A in solving the Chaotic Map Discrete Logarithm Problem (CMDLP) within a runtime of rt is considered negligible if it satisfies $Adv_{A}^{CMDLP}\left(rt\right)<\epsilon$, where ϵ is a negligible function.*

**Theorem** **1.** *For the proposed protocol P, the adversary A operates within polynomial time, aiming to compromise semantic security. Let $q_{h}$ denote the number of hash queries, $q_{p}$ the number of PUF queries, and $q_{s}$ the number of send queries. Additionally, let l represent the length of the biometric data, D be the uniformly distributed password dictionary, and the PUF has a key length of |PUF|. Combining the aforementioned definitions, $Adv_{A}^{CMDLP}\left(rt\right)$ denotes the advantage of solving the CMDLP problem within time rt. Thus, we have:*

$$\begin{array}{cc} Adv_{A}^{P}\;\leq & \frac{q_{h}^{2}}{\left|Hash\right|}+\frac{q_{p}^{2}}{\left|PUF\right|}+2\left(\frac{q_{s}}{2^{l}\cdot \left|D\right|}\;+\;Adv_{A}^{CMDLP}\left(rt\right)\right). \end{array}$$

We establish the provable security of the proposed protocol by defining a series of games $Game_{i}$(i=0,1,2,…,5). Specifically, in our protocol *P*, let $\mathrm{Pr}[{\mathrm{suc}}_{i}]$ denote the event where the adversary A successfully guesses the value of *b* in the Test query within the game $Game_{i}$.

${\mathrm{Game}}_{0}.$ This game models an attack scenario where the adversary A targets the protocol *P*. At the start, A is tasked with determining the value of the bit *b*. The advantage of A in this context is given by:

(1) $${Adv}_{A}^{P}=\left|2\mathrm{Pr}[{\mathrm{suc}}_{0}]-1\right|,$$

where $\mathrm{Pr}[{\mathrm{succ}}_{0}]$ represents the probability of A succeeding in guessing *b*.

${\mathrm{Game}}_{1}.$ In this game, A simulates an eavesdropping attack by performing multiple Execute queries. Assume A intercepts all messages $Msg_{1}$ through $Msg_{4}$ transmitted within the protocol. To determine the session key $SK_{j-i}=h(PID_{i}∥ID_{j}∥psk∥N_{u}∥N_{j})$, A must have access to specific long-term and short-term secret parameters. However, based on the informal security analysis, obtaining these parameters is impractical for A. Therefore, the probability of A successfully winning ${\mathrm{Game}}_{1}$ remains unchanged. Consequently, we have $\mathrm{Pr}\left[{\mathrm{suc}}_{0}\right]=\mathrm{Pr}\left[{\mathrm{suc}}_{1}\right]$.

${\mathrm{Game}}_{2}.$ The primary distinction between $Game_{2}$ and its predecessor, $Game_{1}$, lies in the incorporation of simulated Send and Hash queries. In $Game_{2}$, A performs an active attack, trying to deceive a participant into accepting forged messages. Although the adversary may conduct multiple hash queries on $Msg_{1}$, $Msg_{2}$, $Msg_{3}$, and $Msg_{4}$ to identify potential collisions, the inclusion of random nonces, unique identifiers ($PID_{i}$ and $ID_{j}$), and long-term secrets associated with each message renders this a highly improbable event. As a result, the likelihood of the adversary encountering a collision during Send queries is negligible. Moreover, applying the birthday paradox reinforces this assertion by indicating that

(2) $$\left|\mathrm{Pr}\left[{\mathrm{suc}}_{1}\right]-\mathrm{Pr}\left[{\mathrm{suc}}_{2}\right]\right|\leq \frac{q_{h}^{2}}{2|Hash|}$$

${\mathrm{Game}}_{3}.$ In $Game_{3}$, we simulate PUF queries. Applying the definition of secure PUF functions (Definition 2), we derive the following inequality:

(3) $$\left|\mathrm{Pr}\left[{\mathrm{suc}}_{2}\right]-\mathrm{Pr}\left[{\mathrm{suc}}_{3}\right]\right|\leq \frac{q_{p}^{2}}{2|PUF|}$$

${\mathrm{Game}}_{4}.$ The transition from $Game_{3}$ to $Game_{4}$ involves the addition of the CorruptSmartcard(Ω) query. By utilizing this query, the adversary A will acquire the credentials $TID_{i},X_{i},Y_{i},$ and $Auth_{i}$. To correctly identify $ID_{i}$ and $PW_{i}$ for $U_{i}$ from ($s_{i}^{\prime }\left\|n_{i}^{\prime }\right)=X_{i}⊕h\left(ID_{i}^{\prime }\|PW_{i}^{\prime }\right)$ and $Bk_{i}^{\prime }=\mathrm{Rep}\left(B_{i}^{\prime },s_{i}^{\prime }\right)$, A must have both the secret credential $n_{i}^{\prime }$ and the biometric key $Bk_{i}^{\prime }$. By capping the number of unsuccessful identity/password or biometric verifications, the system upholds the following condition:

(4) $$\left|\mathrm{Pr}\left[{\mathrm{suc}}_{3}\right]-\mathrm{Pr}\left[{\mathrm{suc}}_{4}\right]\right|\leq \frac{q_{s}}{2^{l}\cdot \left|D\right|}$$

${\mathrm{Game}}_{5}.$ The final game involves A endeavoring to compute the shared session key $SK_{i-j}$ between $U_{i}$ and $SD_{j}$ by exploiting intercepted communications and simultaneously solving the CMDLP instance (cf. Definition 3). To calculate the session key $SK_{j-i}=h(PID_{i}∥ID_{j}∥psk∥N_{u}∥N_{j})$, A needs $ID_{j}$ and psk, where $psk=T_{n}\left(T_{m}\left(PID_{i}\right)\right)=T_{m}\left(T_{n}\left(PID_{i}\right)\right)=T_{mn}\left(PID_{i}\right)\;\mathrm{mod}\;lp$. It is evident that even with knowledge of $PID_{i}$, computing $T_{n}\left(PID_{i}\right)\;\mathrm{mod}\;lp$ necessitates access to *n*. Similarly, determining $T_{m}\left(PID_{i}\right)\;\mathrm{mod}\;lp$ requires knowledge of *m*, despite possessing $PID_{i}$. Consequently, A must solve the CMDLP within a runtime of at most rt to obtain the session key $SK_{i-j}$. Therefore,

(5) $$\left|\mathrm{Pr}\left[{\mathrm{suc}}_{4}\right]-\mathrm{Pr}\left[{\mathrm{suc}}_{5}\right]\right|\leq Adv_{A}^{CMDLP}\left(rt\right)$$

Taking into account all the games described earlier, after all the oracle queries have been executed, the adversary A does not gain any extra advantage in correctly guessing the bit *b* in the Test query. Consequently, $\mathrm{Pr}\left[{\mathrm{suc}}_{5}\right]=\frac{1}{2}$. Additionally, based on the calculations, we get:

(6) $$\begin{array}{cc} \left|\mathrm{Pr}\left[{\mathrm{suc}}_{0}\right]-\mathrm{Pr}\left[{\mathrm{suc}}_{5}\right]\right| & =\left|\mathrm{Pr}\left[{\mathrm{suc}}_{0}\right]-\frac{1}{2}\right|=\left|\mathrm{Pr}\left[{\mathrm{suc}}_{1}\right]-\frac{1}{2}\right|\\ \; & \leq \left|\mathrm{Pr}\left[{\mathrm{suc}}_{1}\right]-\mathrm{Pr}\left[{\mathrm{suc}}_{2}\right]\right|\\ \; & \;+\left|\mathrm{Pr}\left[{\mathrm{suc}}_{2}\right]-\mathrm{Pr}\left[{\mathrm{suc}}_{3}\right]\right|\\ \; & \;+\left|\mathrm{Pr}\left[{\mathrm{suc}}_{3}\right]-\mathrm{Pr}\left[{\mathrm{suc}}_{4}\right]\right|\\ \; & \;+\left|\mathrm{Pr}\left[{\mathrm{suc}}_{4}\right]-\mathrm{Pr}\left[{\mathrm{suc}}_{5}\right]\right|\\ \; & =\frac{q_{h}^{2}}{2|Hash|}+\frac{q_{p}^{2}}{2|PUF|}\\ \; & \;+\frac{q_{s}}{2^{l}\cdot \left|D\right|}+Adv_{A}^{CMDLP}\left(rt\right) \end{array}$$

Combining the results yields:

(7) $$\begin{array}{cc} Adv_{A}^{P}= & |2\mathrm{Pr}\left[{\mathrm{suc}}_{0}\right]-1|=2|\mathrm{Pr}\left[{\mathrm{suc}}_{0}\right]-\frac{1}{2}|\\ \leq & \frac{q_{h}^{2}}{\left|Hash\right|}+\frac{q_{p}^{2}}{\left|PUF\right|}+2\left(\frac{q_{s}}{2^{l}\cdot \left|D\right|}+Adv_{A}^{CMDLP}\left(rt\right)\right) \end{array}$$

## 6. Performance Evaluation

In this section, we compare the proposed protocol with five state-of-the-art schemes [60,61,62,63,64], focusing on computational overhead, communication overhead, and security and functionality features.

### 6.1. Computational Overhead

This section analyzes the computational cost of our proposed protocol compared to existing ones. Core cryptographic operations underpinning user login and authentication are considered. We exclude basic operations like XOR and concatenation from the analysis. For the efficiency evaluation, we implemented all cryptographic primitives involved in the proposed protocol and the baseline protocols using Python 3.9.13, leveraging libraries such as *hashlib*, *pypuf*, and *ecpy*. We then measured their average execution times, including hash function ($T_{H}$), ECC point multiplication ($T_{\mathrm{EM}}$), PUF ($T_{\mathrm{PUF}}$), fuzzy extractor ($T_{\mathrm{FE}}\approx T_{\mathrm{EM}}$), and chaotic map ($T_{\mathrm{CM}}$). In addition, to support the testing procedures, a Raspberry Pi 4 equipped with 2 GiB of memory and running Raspberry Pi OS (32-bit) was used as the resource-constrained Platform I to simulate smart sensing devices, whereas a 64-bit Windows 10 machine with 8 GiB of memory and an Intel^®^ Core™ i5-8300H CPU @ 2.30GHz was employed as the resource-rich Platform II to emulate gateway nodes and user devices. For each platform and cryptographic primitive, 1000 test runs were conducted. The resulting average execution times in milliseconds are tabulated in Table 4.

Table 5 summarizes the computational overheads of different protocols. Our protocol incurs a computation overhead of approximately $6T_{H}+T_{\mathrm{PUF}}+T_{\mathrm{FE}}+2T_{\mathrm{CM}}\approx 6.7955$ on resource-constrained smart sensing devices (Platform-I). This is lower than the overhead of protocols proposed by the benchmark protocols of Hammad et al. [60], Wazid et al. [61], and Sutrala et al. [63]. This indicates better suitability for resource-constrained settings. While the total computation overhead of our protocol (8.5445 ms) exceeds that of some existing solutions Yang et al. [62] and Srinivas et al. [64], it offers superior security and functionality features, as detailed in Table 5. This trade-off between efficiency and security is a crucial consideration for real-world deployments.

### 6.2. Runtime Comparison

**Figure 3:**
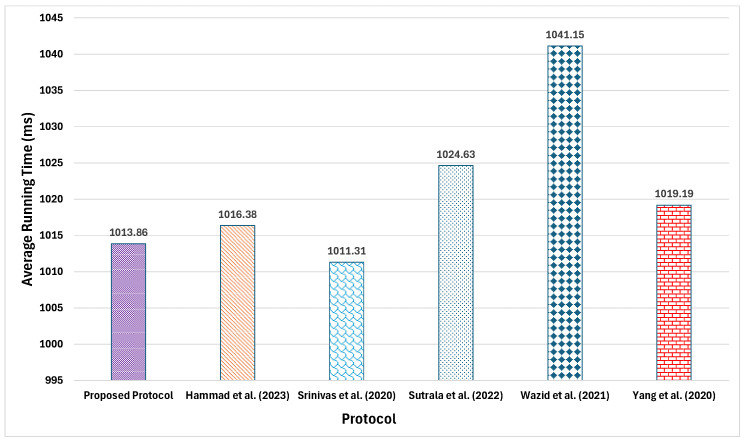
Comparison of computation overheads.

In this subsection, a comprehensive performance evaluation is conducted by implementing the complete workflows of both the proposed protocol and its baseline protocols in Python on a Windows 10 experimental machine equipped with an Intel^®^ Core™ i5-8300H CPU @ 2.30GHz processor and 8 GiB of memory. Each protocol underwent 100 runs, and the average execution time for the integrated login and AKA phase is recorded; comparative results appear in Figure 3. Empirical data indicate that the proposed protocol attains an average runtime of 1013.86 ms, outperforming the protocols of Hammad et al. [60] (1016.38 ms), Sutrala et al. [63] (1024.63 ms), Wazid et al. [61] (1041.15 ms), and Yang et al. [62] (1019.19 ms). Although its overhead is marginally higher than that of the protocol proposed by Srinivas et al. [64], this slight increase is offset by the additional security features and functional enhancements delivered by the proposed protocol (see Table 8).

### 6.3. Communication Overhead

Table 6 presents a comparison of the communication overhead of various protocols, highlighting the number of bits needed for message exchanges. The communication overhead is quantified in bits for each protocol. To ensure a fair evaluation, the following assumptions are made: timestamps are considered to be 32 bits; identities, random numbers, chaotic map-based encryption outputs, and PUF responses are considered to be 128 bits; hash digest outputs are 256 bits; and ECC points are 320 bits. The analysis reveals that the total communication overhead of the proposed protocol is 2944 bits, which is lower than the 3616 bits, 3360 bits, 5376 bits, and 3200 bits required by the benchmark protocols Hammad et al. [60], Wazid et al. [61], Yang et al. [62], and Sutrala et al. [63], respectively. Thus, the proposed protocol is more resource-efficient. Moreover, while the proposed protocol’s communication overhead is slightly higher than that of Srinivas et al. [64], this increase is justified by its enhanced and comprehensive security features, as outlined in Table 8.

### 6.4. Storage Overhead

Building on the experimental configuration in Section 6.3, Table 7 presents a systematic comparison of the storage overhead incurred by the proposed protocol versus baseline protocols. The focus is on the secret credentials that user devices, gateways/servers, and smart sensing units must store after initialization and registration to support subsequent AKA operations. To illustrate scalability, Table 7 also presents the storage cost of each entity as a function of the number of smart sensing devices, denoted by *N*. For resource-constrained smart sensing devices, the proposed protocol requires only 384 bits of local storage-substantially less than the 1280 bits of Wazid et al. [61] and the 1984 bits of Sutrala et al. [63], and equal to the requirements of Srinivas et al. [64] and Yang et al. [62]. Given the enhanced security and functionality delivered by our protocol (see Table 8), this minimal storage footprint is justified. From a system-wide perspective, the total storage burden introduced by the proposed protocol grows linearly as 1024N+2016 bits-markedly lower than Wazid et al.’s [61] 1792N+4320 bits, Yang et al.’s [62] 1152N+1280 bits, and Sutrala et al.’s [63] 2624N+4576 bits. While slightly higher than the schemes of Hammad et al. [60] and Srinivas et al. [64], the proposed protocol offers stronger security guarantees and richer functionality (refer to Table 8), making this trade-off reasonable and acceptable.

**Table 8:** Security and functionality features comparison.

Feature	[60]	[61]	[62]	[63]	[64]	Our
$F_{\infty }$	✓	×	×	×	×	✓
$F_{\in }$	✓	✓	×	✓	✓	✓
$F_{∋}$	✓	✓	✓	✓	✓	✓
$F_{△}$	✓	✓	✓	✓	✓	✓
$F_{▽}$	✓	✓	✓	✓	✓	✓
$F_{/}$	✓	✓	✓	✓	✓	✓
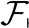	✓	✓	✓	✓	✓	✓
$F_{\forall }$	×	✓	✓	✓	✓	✓
$F_{\exists }$	✓	✓	✓	✓	✓	✓
$F_{\infty \prime }$	×	×	✓	×	×	✓
$F_{\infty \infty }$	✓	✓	✓	✓	✓	✓
$F_{\infty \in }$	✓	✓	✓	✓	✓	✓
$F_{\infty ∋}$	✓	✓	✓	✓	✓	✓

**Note:** $F_{\infty }$: Smart sensing device capture attack; $F_{\in }$: Anonymity; $F_{∋}$: Untraceability; $F_{△}$: De-synchronization attack; $F_{▽}$: Replay attack; $F_{/}$: MitM attack; 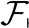: Mutual authentication; $F_{\forall }$: Independent session key establishment; $F_{\exists }$: Perfect forward secrecy; $F_{\infty \prime }$: No clock synchronization; $F_{\infty \infty }$: ESL attack; $F_{\infty \in }$: Stolen smartcard and privileged-insider attacks; and $F_{\infty ∋}$: Impersonation attacks; ✓: indicates feature availability; ×: indicates feature unavailability or inapplicability.

### 6.5. Security and Functionality Features

Table 8 presents a comparison of the proposed protocol against the benchmark protocols: Hammad et al. [60], Wazid et al. [61], Yang et al. [62], Sutrala et al. [63], and Srinivas et al. [64]. The comparison evaluates thirteen security and functionality features: $F_{\infty }$: Smart sensing device capture attack; $F_{\in }$: Anonymity; $F_{∋}$: Untraceability; $F_{△}$: De-synchronization attack; $F_{▽}$: Replay attack; $F_{/}$: MitM attack; 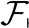: Mutual authentication; $F_{\forall }$: Independent session key establishment; $F_{\exists }$: Perfect forward secrecy; $F_{\infty \prime }$: No clock synchronization; $F_{\infty \infty }$: ESL attack; $F_{\infty \in }$: Smartcard theft and insider threats; and $F_{\infty ∋}$: Impersonation attacks. In Table 8, a check mark (✓) denotes the presence of a feature, while a cross (×) indicates its absence or inapplicability. The comparison reveals that the proposed protocol is the only one to encompass all the essential and critical security and functionality features. Conversely, the benchmark protocols show shortcomings, either missing certain features or failing to counter specific security threats.

### 6.6. Critical Discussion

The proposed AKA protocol delivers a balanced solution for real-time IIoT environments by integrating PUFs, fuzzy extractors, and chaotic maps, ensuring strong resistance to attacks and meeting all targeted security and functionality objectives. It maintains moderate computational and communication overheads, making it suitable for resource-constrained devices, with superior runtime performance. Limitations include PUF reliability under environmental variations, computational overhead from fuzzy extractors, and reliance on simulations lacking real-world insights. Trade-offs involve latency, storage overhead, and implementation complexity. Future work targets real-world testbed implementation, IIoT communication stack integration, robust key management, recovery mechanisms, and energy efficiency evaluation under diverse scenarios.

## 7. Conclusions

To address the threats posed by various known attacks on wireless data communication between users, gateway nodes, and smart sensing devices in Industrial Internet of Things (IIoT) network scenarios, this paper proposes an anonymous and secure authentication and key agreement protocol for IIoT settings. The proposed protocol is based on PUF, cryptographic hash, XOR, and Chaotic Map, providing strong security features while maintaining resource efficiency, making it more suitable for resource-constrained IIoT environments. Furthermore, a comprehensive comparison with existing protocols demonstrates that the proposed protocol significantly reduces computational and communication overhead while offering superior security features, representing a substantial advancement in the field. Future work includes the exploration of post-quantum cryptographic primitives to ensure long-term resistance against quantum-capable adversaries, and the experimental deployment of the proposed protocol in real-world IIoT testbeds to validate its performance under diverse and dynamic environmental conditions.

## Figures and Tables

**Table 1:** Existing Authentication Protocols: A Comparative Summary.

Protocol	Cryptographic Primitives	Descriptions	Limitations
Turkanović et al. [21]	• Cryptographic hash function	• Lightweight • Dynamic addition of sensor nodes • Free password update	• No PUF-based physical security provided • Strict clock synchronization required • Anonymity not provided • No resistance to sensor capture attacks
Chen et al. [23]	• Cryptographic hash function • Elliptic curve cryptography	• Supporting node revocation • No strict clock synchronization	• No PUF-based physical security provided • Excessive system overhead • Lack of perfect forward secrecy • No resistance to ephemeral secret leakage attacks
Shuai et al. [24]	• Cryptographic hash function • Rabin cryptosystem	• Free password update • Dynamic addition of sensor nodes	• No PUF-based physical security provided • Strict clock synchronization required • No resistance to offline guessing and user impersonation attacks • No resistance to privileged insider and eavesdropping attacks • No resistance to stolen smart-card attacks
Zhai et al. [28]	• Cryptographic hash function • Chaotic Map	• Lightweight • Supporting cross-domain authentication	• No PUF-based physical security provided • Strict clock synchronization required • Lack of anonymity and untraceability
Aman et al. [29]	• Cryptographic hash function • PUF	• Lightweight • Providing PUF-based physical security • No strict clock synchronization	• No resistance to replay and non-invasive physical attacks • The neglect of PUF noise impact
Rafique et al. [31]	• Cryptographic hash function • AES symmetric encryption/decryption	• Lightweight	• No PUF-based physical security provided • No resistance to smart-card and device theft attacks • Strict clock synchronization required
Eldefrawy et al. [33]	• Cryptographic hash function	• Lightweight • Free password update • Dynamic addition/revocation of sensor nodes • No strict clock synchronization	• No PUF-based physical security provided • Lack of mutual authentication
Harishma et al. [34]	• Cryptographic hash function • Identity-based encryption • PUF	• Providing PUF-based physical security • No strict clock synchronization	• No resistance to ephemeral secret leakage attacks
Chen et al. [36]	• Cryptographic hash function • Elliptic curve cryptography	• Lightweight	• No PUF-based physical security provided • No resistance to insider and node-capturing attacks • Strict clock synchronization required • Lack of untraceability

**Table 2:** Symbols and their definitions.

Symbol	Definition
$GWN_{k}$	*k*th GWN
$X_{GWN_{k}}$	Secret parameter of $GWN_{k}$
$ID_{GWN_{k}}$	Identity of $GWN_{k}$
$SD_{j}$	*j*th smart sensing deivce
$C_{j}$	Challenge component for $SD_{j}$
$DR_{j}$	Response component for $SD_{j}$
$X_{j}$	Secret parameter bound to $SD_{j}$
$Dk_{j},s_{j}$	Cryptographic key and auxiliary data of $SD_{j}$
$U_{i}$	*i*th user
$ID_{i},PW_{i}$	Identity and password of $U_{i}$
$B_{i}$	Biometric template of $U_{i}$
$Bk_{i},s_{i}$	Cryptographic key and auxiliary data of $U_{i}$
$SC_{i},SCN_{i}$	Smart card of $U_{i}$ and its number of $U_{i}$
$X_{i},Y_{i},Auth_{i}$	Secret parameter bound to $U_{i}$
$PID_{i}$	Pseudo-identity of $U_{i}$
$PUF_{j}\left(\cdot \right)$	Physical unclonable function associated with $SD_{j}$
RA	The trusted registration authority
SCN	Smart card number
SK	Session key
$TID_{i}$	Temporary identity of $U_{i}$
$TID_{i}^{o}/TID_{i}^{n}$	The Old/new temporary identitity
$X_{RA}$	Secret key of RA
h(·)	Cryptographic hash function
Gen(·)	Fuzzy extractor generation function
Rep(·)	Fuzzy extractor reproduction function

**Table 3:** Adversary queries in the ROR model.

Query	Functionality
** $Execute(\Omega_{U_{i}}^{t_{1}},\Omega_{GWN_{k}}^{t_{2}},\Omega_{SD_{j}}^{t_{3}})$ **	This query allows the adversary A to passively observe and collect messages exchanged among $\Omega_{U_{i}}^{t_{1}},\Omega_{GWN_{k}}^{t_{2}},$ and $\Omega_{SD_{j}}^{t_{3}}$, facilitating the evaluation of forward secrecy.
** $Send(\Omega^{t},msg)$ **	This query enables the simulation of active attacks, such as impersonation and replay. The adversary A sends a message msg to $\Omega^{t}$ and receives the corresponding response.
** $CorruptSmartcard(\Omega^{t})$ **	Upon executing this query, the adversary A obtains complete access to the credentials stored on the compromised smart card $SC_{i}$ belonging to the legitimate registered user $U_{i}$.
** $Reveal(\Omega_{U_{i}}^{t_{1}})$ **	This query discloses the actual session key if $\Omega_{U_{i}}^{t_{1}}$ has been accepted.
** $Test(\Omega^{t})$ **	This query returns the real session key when b=1, or a random string of the same length when b=.. If A can consistently guess the value of *b* correctly, it signifies a successful compromise of the session key’s semantic security.

**Table 4:** Average execution time of various primitives.

Operation	Platform-I	Platform-II
Hash function ($T_{H}$)	0.007 ms	0.001 ms
ECC point multiplication ($T_{\mathrm{EM}}$)	6.549 ms	0.846 ms
ECC point addition ($T_{\mathrm{EA}}$)	0.273 ms	0.002 ms
Physical unclonable function ($T_{\mathrm{PUF}}$)	0.5 μs	–
Fuzzy extractor ($T_{\mathrm{FE}}\approx T_{\mathrm{EM}}$)	6.549 ms	0.846 ms
Chaotic map ($T_{\mathrm{CM}}$)	0.102 ms	0.019 ms

**Table 5:** Computation overheads comparison (ms).

Protocol	User	GWN / Server	Smart Sensing Device	Total Overhead
Hammad et al. [60]	$T_{PUF}+2T_{EM}+7T_{H}\approx 1.699$	$2T_{EM}+9T_{H}\approx 1.701$	$T_{PUF}+2T_{EM}+5T_{H}\approx 13.1335$	16.5335
Wazid et al. [61]	$T_{FE}+4T_{EM}+T_{EA}+19T_{H}\approx 4.251$	$5T_{EM}+T_{EA}+T_{H}\approx 4.233$	$4T_{EM}+T_{EA}+12T_{H}\approx 26.553$	35.037
Yang et al. [62]	$10T_{H}\approx 0.01$	$19T_{H}\approx 0.019$	$8T_{H}\approx 0.056$	0.085
Sutrala et al. [63]	$T_{FE}+5T_{EM}+2T_{EA}+16T_{H}\approx 5.096$	$3T_{EM}+2T_{EA}+9T_{H}\approx 2.551$	$4T_{EM}+T_{EA}+8T_{H}\approx 26.525$	34.172
Srinivas et al. [64]	$15T_{H}+T_{\mathrm{FE}}+2T_{\mathrm{CM}}\approx 0.899$	$10T_{H}\approx 0.01$	$6T_{H}+2T_{\mathrm{CM}}\approx 0.246$	1.155
Our Proposed	$8T_{H}+T_{\mathrm{FE}}+2T_{\mathrm{CM}}\approx 0.892$	$11T_{H}+T_{\mathrm{FE}}\approx 0.857$	$6T_{H}+T_{\mathrm{PUF}}+T_{\mathrm{FE}}+2T_{\mathrm{CM}}\approx 6.7955$	8.5445

**Table 6:** Communication overheads comparison.

Protocol	No. of Messages	Total Overhead (Bits)
Hammad et al. [60]	5	3616
Wazid et al. [61]	3	3360
Yang et al. [62]	6	5376
Sutrala et al. [63]	3	3200
Srinivas et al. [64]	3	1856
Our Proposed	4	2944

**Table 7:** Comparison of storage overheads (bits).

Protocol	User	GWN/Server	Smart Sensing Device	Overall Overheads
Hammad et al. [60]	640	1024+128n	512	640N+2176
Wazid et al. [61]	256N+2656	256N+1664	1280	1792N+4320
Yang et al. [62]	128N+640	640N+640	384	1152N+1280
Sutrala et al. [63]	256N+2592	384N+1984	1984	2624N+4576
Srinivas et al. [64]	128N+992	384N+128	384	896N+1120
Our Proposed	128N+1120	640N+896	384	1024N+2016

Note: *N*: Number of Smart Sensing Devices.

## Data Availability

Data are available from the authors upon reasonable request.
